# Translational Research in Pharmaceutical Sciences

**Published:** 2018

**Authors:** Jamshid Salamzadeh


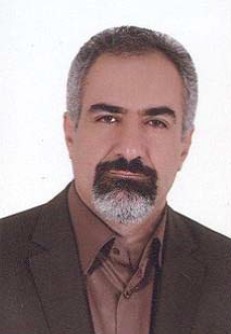


Different branches of science are expanding very fast and there are vast volumes of new information which are progressively discovered and added to the present human knowledge. With no doubt, pharmaceutical sciences is amongst the most dynamic disciplines of science that its content is attributed to different basic and applied researches including studies in the field of physical and organic chemistry, engineering, biochemistry, biology, pharmacology and pharmacotherapy. These researches are aimed to understand how to develop new drugs, optimize their delivery to the body and translate these integrated understanding into new therapies against human disease as well as improved community health. 

Although in recent past, the main emphasize was to integrate advancements in biomedical field with clinical practice, *i.e.* taking research from the “bench” to ‘bedside”, however, nowadays, the “translational medicine” put emphasize on translation of basic scientific findings in bench-side into potential treatments for disease and to enhance public health, where a combination of disciplines, resources, expertise, and techniques within the three main pillars of “bench-side”, “bedside” and “community” should be applied to endorse developments in prevention, diagnosis, and therapies of diseases. This is where economical evaluations and commercialization are also important parts of the translational research.

In the field of pharmaceutical research, there are bidirectional inter- and intra-disciplinary relationships between and within main pillars of the translational research. Molecular and pre-clinical bench-based information attained in the laboratory along with those findings obtained by human and clinical studies need to be understood and scientifically interpreted as pooled evidence. These should eventually be integrated with information being feed-backed by community-based clinical practice leading to more efficient discoveries benefitting health care systems. Clinical observations about the nature, pathogenesis and development of diseases as well as responses to and complications caused by therapies can effectively drive basic science investigations. Knowledge obtained by basic science then provides clinical practitioners with new treatment strategies. This continuous translational research cycle can hopefully result in more rational drug design, improved efficacy of therapeutic agents, and accelerated optimization of investigational compounds for clinical use, which can finally be used in the public health sphere. Besides, this will lead to a more cost-effective drug discovery process.

In conclusion, a hub for bench-to-bedside-to-community is required for conducting the pharmaceutical researches towards more efficient applied outcomes improving public health. This requires a structured, well-organized, reproducible and continuous relationship between three main backbones of translational research i.e. bench-side, bedside and community.

*Jamshid Salamzadeh is Professor in Department of Clinical Pharmacy, and Pharmacoeconomy and Pharma-management, School of Pharmacy, Shahid Beheshti University of Medical Sciences, Tehran, Iran. He is also president of the Iranian Society of Clinical Pharmacists.*

*e-mail address: j.salamzadeh@sbmu.ac.ir*

